# A Classical Formulation of Quantum Theory?

**DOI:** 10.3390/e24010137

**Published:** 2022-01-17

**Authors:** William F. Braasch, William K. Wootters

**Affiliations:** 1 Department of Physics and Astronomy, Dartmouth College, Hanover, NH 03755, USA; wbraasch@gmail.com; 2 Department of Physics, Williams College, Williamstown, MA 01267, USA

**Keywords:** Wigner function, qubit, quasiprobability, epistemic restriction, quantum reconstruction, phase space

## Abstract

We explore a particular way of reformulating quantum theory in classical terms, starting with phase space rather than Hilbert space, and with actual probability distributions rather than quasiprobabilities. The classical picture we start with is epistemically restricted, in the spirit of a model introduced by Spekkens. We obtain quantum theory only by combining a *collection* of restricted classical pictures. Our main challenge in this paper is to find a simple way of characterizing the allowed sets of classical pictures. We present one promising approach to this problem and show how it works out for the case of a single qubit.

## 1. Introduction

Much of Wojciech Zurek’s research, including his research on quantum Darwinism, has been aimed at explaining the emergence of the classical world from the quantum world. This is of course an important endeavor, partly because, as he has pointed out, quantum theory and classical physics seem almost incompatible at first sight.

“The quantum principle of superposition implies that any combination of quantum states is also a legal state. This seems to be in conflict with everyday reality: States we encounter are localized. Classical objects can be either here or there, but never *both* here and there”

[1].

Indeed, it is an interesting fact that the standard formulation of quantum theory—with state vectors in Hilbert space—looks as different as it does from the emergent classical picture. In this paper, we take a step towards a reformulation of quantum theory that looks more classical from the very beginning, being based on phase space rather than Hilbert space. At the same time, we wish to avoid the negative probabilities of the Wigner-function formulation, which is the most common phase-space formulation of quantum theory.

We are motivated largely by the general observation that it is good to have alternative formulations of a well-established theory. Alternative formulations can provide novel insights and new methods of analysis. In the present case, we can also hope that our classical-like formulation will ultimately provide another perspective on the quantum-to-classical transition.

Though we intend in future work to apply our methods to continuous quantum variables such as position and momentum, in this paper, we restrict our attention to the case of systems normally described with a finite-dimensional Hilbert space. For us, this means that the phase spaces we use are *discrete*. Specifically, we use the discrete phase space introduced in [2], which is simplest when the Hilbert-space dimension *d* is prime. In that case, the phase space is a d×d array of points, with axes—analogous to position and momentum axes—labeled by elements of the field $Z_{d}$, that is, the integers mod *d*. As we explain in the following section, in this phase space it makes sense to speak of “lines” and “parallel lines”. Each line has exactly *d* points, and there are d+1 ways of dividing the $d^{2}$ points of phase space into *d* parallel lines.

Our work is related to a construction due to Spekkens [3,4,5,6]. Starting with the same discrete phase space, he defines an “epistemically restricted classical theory”: the points of phase space are understood to be the actual, underlying states of the system, but an observer cannot know this state. The most detailed description an observer can give is a uniform probability distribution over one of the lines. Spekkens showed that many qualitative features of quantum theory can be captured by this model, but the model cannot fully imitate quantum theory because it is non-contextual.

In a recent paper, we showed how one can construct a picture that borrows some of Spekkens’ ideas but that accommodates the full quantum theory of a *d*-state system [7]. Specifically, we found that one can decompose the quantum description of a complete experiment—a preparation, a transformation (or a sequence of transformations), and a measurement—into a collection of classical descriptions, each entailing certain epistemic restrictions similar to but subtly different from the one imposed in Spekkens’ model. There is one such classical description for each possible choice of what we call a “framework.” The framework defines the epistemic restrictions placed on the classical model. Within each framework, we can imagine a classical observer whose picture of the experiment is perfectly compatible with an ontological model in which the system really does occupy a definite phase-space point at every moment, and in which a transformation is represented by an ordinary set of transition probabilities in phase space. Each classical observer will compute their own prediction for the experiment, in the form of a probability assigned to each possible outcome. We showed how to combine these classical predictions to reconstruct the quantum prediction. In a slogan, we say the quantum prediction is obtained by “summing the nonrandom parts”. The meaning of this slogan will become clear in the following section, but essentially, the nonrandom part of a probability value is its deviation from the value one would use under a condition of minimal knowledge. (Thus the expression, “the nonrandom part,” is a kind of shorthand. We do not mean to imply that there is no element of randomness in values of a probability that differ from the minimal-knowledge value.) Intriguingly, the use of this unusual method of combining probability distributions allows us to reproduce the operational statistics of the non-commutative theory of quantum mechanics starting with ordinary (commutative) classical probability theory.

In [7], we were not able to come up with a simple set of criteria for determining precisely what sets of classical descriptions are allowed—we did specify a set of criteria, but it is not simple. Such criteria are desirable if our formulation of quantum theory is to be self-contained, that is, not dependent on concepts from the Hilbert-space formulation. The primary aim of the present paper is to identify such a set of criteria.

The rest of this paper is organized as follows. In the next section, we review the formalism of [7]: how the frameworks are defined, how one decomposes the quantum description of an experiment into epistemically restricted classical descriptions, and how the predictions based on these classical descriptions are combined to recover the quantum prediction. In Section 3, we write down four equations showing how any pair of components of an experiment—the components being preparations, transformations, and measurement outcomes—can be combined to obtain either other components or an observable probability. For example, a preparation followed by a transformation constitutes another preparation, and for a preparation followed directly by a yes-or-no test, there is an equation that yields the probability of the outcome “yes”. The basis of each of these four equations is the principle that the nonrandom parts of the inputs should be summed to obtain the nonrandom part of the output. At this point, we ask our main question: To what extent do these four composition rules determine the allowed sets of classical descriptions? That is, to what extent do these equations characterize the structure of quantum theory for the *d*-state system? We find it useful to add a few auxiliary assumptions, but we do not know whether all these assumptions are necessary. Conceivably, a more parsimonious set of postulates is possible.

In Section 4, we specialize to the case of a single qubit and ask whether the composition rules and auxiliary postulates of Section 3 determine the quantum theory of this simple system. We find that we recover either the standard quantum theory for a qubit or a theory with a discrete set of transformations.

Of course, we would like to extend this approach to all possible Hilbert-space dimensions and to composite systems. We discuss the possibilities for doing this in the concluding section.

For the remainder of this Introduction, we review briefly some of the earlier efforts to reconstruct quantum theory from basic principles, as well as other work on quantum theory in phase space and other approaches to representing quantum theory in terms of probability distributions.

Reconstructions of quantum theory can be traced back to Birkhoff and von Neumann [8]. In these initial forays, the focus was on mathematical axiomatizations [9,10,11,12]. However, it is appealing to think that quantum mechanics might be reconstructed by stipulating a set of principles in the spirit of Einstein’s principles that lead to the theory of special relativity. This more operationally oriented approach was ignited by Hardy [13]. In Hardy’s axiomatization, the addition of the key word “continuous” to one of his principles differentiates quantum mechanics from classical probability theory. Although the approach we describe here is different from Hardy’s, that same key word rears its head as the distinguishing feature between quantum mechanics and a simpler theory, as we will see in Section 4. Other important reconstruction efforts have likewise relied on operational or information-theoretic principles [14,15,16,17,18,19]. Recently, diagrammatic postulates have been used to reconstruct quantum theory [20].

In another vein, attempts to pinpoint essential quantumness have taken the tack of augmenting classical physics with simple rules. As we mentioned, Spekkens and collaborators have done this in a series of epistemically restricted classical theories used to support an epistemic interpretation of the quantum state [3,4,5]. Spekkens’ model has previously been provided with a contextual extension, but without fully capturing quantum theory [21]. It has also been shown to hold strong similarities to stabilizer physics [22,23,24], thereby providing a link to a subtheory of quantum mechanics that plays an important role in quantum computing. Spekkens’ model is naturally set in phase space, which provides the backbone for our work.

Quantum mechanics set in phase space has a history almost as long as quantum mechanics itself [25,26]. Again, the most commonly used phase-space representation of a quantum state is the Wigner function. An interesting complementary strategy is to invert the definition of the Wigner function to make classical mechanics look more like quantum theory [27,28]. A number of different discrete Wigner functions have been defined for finite-dimensional quantum systems [2,29,30,31,32,33]. In this paper, although our aim is to go beyond Wigner functions and use only nonnegative probabilities, we do use concepts from the Wigner-function definition of [2].

There are numerous reasons to study quantum mechanics in phase space. In quantum optics, the appearance of the negativity of the Wigner function signals the onset of quantum behavior [34,35]. The negativity of the Wigner function has also been linked to contextuality, which is another famous notion of nonclassicality [36,37,38], and to the power of quantum computing [24,39,40,41].

The tomography of quantum states is closely tied to the Wigner function. We effectively use tomographic representations of quantum states in this paper. For this reason, our work is also closely related to the “classical” approach to quantum theory found in [42,43,44]. The authors of these papers have successfully described a large number of quantum phenomena from this perspective. Our approach diverges from theirs in that we treat every aspect of a quantum experiment tomographically.

Certain subtheories of quantum mechanics have been proven to be nonnegative in the Wigner-function representation. The stabilizer subtheory is one of them [31], and sampling its nonnegative representation provides the basis for the classical simulation of stabilizer physics [39]. Under certain assumptions, it has been shown that the full quantum theory requires negativity in some aspect (preparation, transformation, or measurement) of a frame-theoretic generalization of the Wigner-function representation [45,46,47]. The Pusey–Barrett–Rudolph theorem is another expression of the limitation on representing quantum mechanics with classical probability theory [48]. Nonetheless, in the search for new ways in which to simulate quantum systems, researchers have found positive probabilistic representations of quantum theory by loosening certain assumptions upon which the theorems are built [49,50,51,52,53,54]. In another setting, Fuchs and Schack have expressed quantum states and transformations as probability distributions over the possible outcomes of a SIC-POVM [55].

Again, our approach begins by decomposing the quantum description of an experiment into a collection of classical probabilistic descriptions, as we explain more fully in the following section.

## 2. A Quantum Experiment as a Collection of Classical Experiments

In this section, we briefly review the formalism developed in [7]. We begin with a bit of notation and terminology.

Let us assume for now that the system we are studying has a Hilbert space with prime dimension *d*. We use Greek letters to label the points of the d×d phase space. Each point α can be specified by its horizontal and vertical coordinates, which we write as $\alpha_{q}$ and $\alpha_{p}$, respectively, to emphasize the analogy with position and momentum. Here $\alpha_{q}$ and $\alpha_{p}$ both take values in $Z_{d}$. A *line* is the set of points α satisfying an equation of the form $a\alpha_{q}+b\alpha_{p}=c$ for fixed $a,b,c\in Z_{d}$ with *a* and *b* not both zero. Two lines are parallel if they can be specified by equations of this form differing only in the value of *c*. We refer to a line passing through the origin as a *ray*.

A point in phase space is not a valid quantum state, but we find it extremely helpful to associate with each point α a quasi-density matrix ${\hat{A}}_{\alpha }$, which we call a “phase point operator.” This is a trace-one Hermitian matrix, but it is not a legitimate density matrix because it can have negative eigenvalues. The matrices ${\hat{A}}_{\alpha }$ that we use are the ones introduced in [2] to define a discrete Wigner function (see below). The matrix ${\hat{A}}_{\alpha }$ for any odd prime *d* is written as follows in terms of its components:

(1) $${\left({\hat{A}}_{\alpha }\right)}_{kl}=\delta_{2\alpha_{q},k+l}\omega^{\alpha_{p}\left(k-l\right)},$$

where $\omega =e^{2\pi i/d}$ and the arithmetic in the subscript of the Kronecker delta is mod *d*. For d=2, there is a special formula:

(2) $${\hat{A}}_{\alpha }=\frac{1}{2}\left[\hat{I}+{\left(-1\right)}^{\alpha_{p}}\hat{X}+{\left(-1\right)}^{\alpha_{q}+\alpha_{p}}\hat{Y}+{\left(-1\right)}^{\alpha_{q}}\hat{Z}\right],$$

where $\hat{X},\hat{Y},\hat{Z}$ are the Pauli matrices and $\hat{I}$ is the 2×2 identity matrix. The $\hat{A}$ matrices are orthogonal in the Hilbert–Schmidt sense:

(3) $$\mathrm{tr}\left({\hat{A}}_{\alpha }{\hat{A}}_{\beta }\right)=d\delta_{\alpha \beta },$$

and because there are $d^{2}$ of them, they serve as a basis for the space of all d×d matrices. In particular, we can expand a density matrix $\hat{w}$ as a linear combination of $\hat{A}$’s.

(4) $$\hat{w}=\sum_{\alpha }Q\left(\alpha |\hat{w}\right){\hat{A}}_{\alpha }.$$

The coefficients $Q(\alpha |\hat{w})$ in this expansion constitute the *discrete Wigner function* representing the given state.

The operators ${\hat{A}}_{\alpha }$ also have the following special property, which we use immediately in the following subsection. For any line *ℓ*, the average of the $\hat{A}$’s over that line is a one-dimensional projection operator:

(5) $$\frac{1}{d}\sum_{\alpha \in \ell }{\hat{A}}_{\alpha }=|\psi_{\ell }\rangle \langle \psi_{\ell }|,$$

where $|\psi_{\ell }\rangle$ is a state vector associated with the line *ℓ*. Since the $\hat{A}$’s are orthogonal to each other, the |ψ〉’s associated with a complete set of parallel lines—we call such a set a striation—constitute an orthonormal basis for the Hilbert space. Moreover, because any two non-parallel lines intersect in exactly one point, Equation (3) guarantees that these bases are mutually unbiased; that is, each basis vector is an equal-magnitude superposition of the vectors of any of the other bases.

### 2.1. Defining the Frameworks

A “framework” is a mathematical structure that determines what epistemic constraint one of our classical probability distributions must satisfy. The introduction of the concept of a framework is one way in which our work differs from Spekkens’ model. In Spekkens’ model, there is just one classical world, and there is one epistemic constraint that applies to it. In his model, for example, a uniform probability distribution over any line of the discrete phase space counts as a legitimate epistemic state. By contrast, what we are doing, roughly speaking, is to decompose this set of possibilities into distinct cases—one for each possible slope of a line—and to associate each of these cases with a different classical world.

We now explain specifically what kind of mathematical structure constitutes a framework for each component of an experiment—a preparation, a transformation, or a measurement—and what epistemic restriction is associated with each of these frameworks.

For either a preparation or a measurement, a framework is simply a striation of the phase space—a complete set of parallel lines. We label such a striation with the symbol *B*, since each striation is associated with an orthonormal basis. For a given framework *B*, the classical probability function representing a given preparation or measurement outcome is required to be constant along each line of the striation *B*—this is the epistemic restriction associated with the framework *B*. We define these restricted probability functions in the next subsection.

To define the framework for a transformation, we need to consider a special class of linear transformations on the discrete phase space. Let us think of a point α as represented by a column vector with components $\alpha_{q}$ and $\alpha_{p}$. Then a linear transformation is represented by a 2×2 matrix, with elements in $Z_{d}$, acting from the left on this column vector. A *symplectic* transformation is a linear transformation that preserves the symplectic product:

(6) $$\langle \alpha ,\beta \rangle =\alpha_{p}\beta_{q}-\alpha_{q}\beta_{p}.$$

For the case we consider, in which the phase space has just two discrete dimensions, the symplectic transformations are the same as the transformations whose matrices have unit determinant.

The number of symplectic matrices for any prime *d* is $d(d^{2}-1)$. Our formulation is simplest if, among these symplectic matrices, there exists a set T of just $d^{2}-1$ such matrices that has the “nonsingular difference” property: the difference between any two matrices in T has a nonzero determinant. It turns out that this condition allows for a particularly simple reconstruction of a quantum transformation from the classical transition probabilities, defined in the following section. In [7], the nonsingular difference is the only property we require of the set T, and to our knowledge, it is not known whether such a set of $d^{2}-1$ matrices exists for all prime *d*. However, for our present purposes, we also need T to constitute a *group*, that is, a subgroup of the symplectic group Sp(2,*d*). It is known that such a special subgroup exists for the values d=2,3,5,7,11 but not for larger values [56]. We can develop our formalism so as to apply to every prime dimension, regardless of whether there exists a special set T—indeed, we did this in [7]. (If no such set exists, we use the full symplectic group and insert a factor of 1/d whenever we sum over the symplectic matrices.) However, in the present paper, for simplicity, we restrict our attention to those dimensions for which such a special subgroup exists. This makes the equations simpler. In Section 4, we specialize to the case of a single qubit, for which we now write down explicitly the unique special subgroup of symplectic matrices:

(7) $$I=\left(\begin{array}{cc} 1 & 0\\ 0 & 1 \end{array}\right)\;R=\left(\begin{array}{cc} 0 & 1\\ 1 & 1 \end{array}\right)\;L=\left(\begin{array}{cc} 1 & 1\\ 1 & 0 \end{array}\right)$$

One can verify that the difference between any two of these matrices has a nonzero determinant. The choice of symbols comes from the fact that R permutes the three nonzero points by rotating them to the right, whereas L rotates them to the left.

Now, finally, we can say what we mean by a framework for a transformation. Again, let T be a group of $d^{2}-1$ symplectic transformations with the nonsingular difference property. Then a framework for a transformation is simply a symplectic matrix *S* chosen from the set T. (When such a group does not exist, we let every symplectic matrix define a framework.)

As we have said, for a preparation or a measurement outcome, the associated probability function—defined in the following subsection—will be required to be constant along each line of the striation *B* serving as the framework. Something similar happens for a transformation. However, instead of working in phase space per se, we now imagine ourselves working in the set of all *ontic transitions* from one point to another. Let us label such a transition as α→β. There are $d^{4}$ ontic transitions. Now, just as a striation *B* partitions the $d^{2}$ points of phase space into *d* sets of *d* points each, we can regard a symplectic transformation *S* as partitioning the $d^{4}$ ontic transitions into $d^{2}$ sets, each comprising $d^{2}$ transitions.

Here is how this partitioning happens. For a fixed symplectic matrix *S* and a given ontic transition α→β, let the *displacement* δ be defined by δ=β−Sα. That is, δ is the extra displacement one needs to arrive at β, once one has applied *S* to α. Keeping *S* fixed, we define the “displacement class” associated with δ to be the set of all the ontic transitions α→β such that β−Sα=δ. For any given *S*, there are $d^{2}$ displacement classes, each consisting of $d^{2}$ ontic transitions. The framework *S* entails the following epistemic restriction: the classical transition probabilities characterizing a given transformation must be *constant* within each displacement class. In the following subsection, we show how such a set of transition probabilities is to be defined.

Consider now an entire experiment consisting of a preparation, a transformation, and a measurement. A framework for the whole experiment is obtained by choosing a framework for each component of the experiment. We express a framework F for this experiment as the ordered triple $F=(B^{\prime },S,B)$, where *B* and $B^{\prime }$ are the frameworks for the preparation and measurement, respectively. (We read the ordered triple from right to left, because in our equations, this is the order in which the associated probability functions will appear.)

As it turns out, we need to consider only a subset of the possible combinations $\left(B^{\prime },S,B\right)$, namely those for which the striation $B^{\prime }$ is precisely the striation obtained by applying *S* to the striation *B*. We call such a combination a *coherent* framework for the experiment. We *may* use other frameworks—ones that are not coherent—but it turns out that such frameworks will contribute nothing to our predictions for the outcome of the experiment. Similarly, if the experiment includes two or more successive transformations, so that we have a framework $\left(B^{\prime },S_{n},\ldots ,S_{1},B\right)$, we need to consider only those frameworks for which $B^{\prime }=S_{n}\ldots S_{1}B$.

We are now ready to show how the quantum description of a preparation, a transformation, or a measurement outcome can be replaced by a set of epistemically restricted classical descriptions.

### 2.2. Decomposing the Quantum Description of an Experiment into Classical Descriptions

We begin with the case of a preparation. The standard quantum description of a preparation is given by a density matrix $\hat{w}$. We replace this single quantum description with d+1 classical descriptions $R^{B}\left(\alpha |\hat{w}\right)$, one for each striation *B*. Each of these classical descriptions is simply a probability distribution over phase space, and each of these probability distributions satisfies the epistemic constraint associated with *B*: the distribution must be constant along each line of *B*.

The definition of $R^{B}\left(\alpha |\hat{w}\right)$ in terms of the density matrix $\hat{w}$ is simple:

(8) $$R^{B}\left(\alpha |\hat{w}\right)=\frac{1}{d}\langle \psi_{\ell }\left|\hat{w}\right|\psi_{\ell }\rangle ,$$

where *ℓ* is the unique line in *B* that contains the point α. Again, $|\psi_{\ell }\rangle$ is the state vector associated with the line *ℓ*. It is not hard to show that *R* is a properly normalized probability distribution over phase space—that is,

(9) $$\sum_{\alpha }R^{B}\left(\alpha |\hat{w}\right)=1,$$

and it is clear that *R* is constant over each line in *B*. It is possible to reconstruct $\hat{w}$ from the whole set of *R*’s, but in this paper, our ultimate aim is to work wholly with the classical descriptions. Therefore, we would like to think of the *R*’s as the primary description of the preparation.

We now move on to the case of a measurement (saving the more complicated case of a transformation for later in this subsection). We are interested just in the probabilities of the outcomes of a measurement, not in any change in the system caused by the measurement. A measurement in this sense is represented in quantum theory by a POVM, that is, a set of positive semidefinite operators on the *d*-dimensional Hilbert space that sum to the identity. Let $\hat{E}$ be one element of such a POVM, corresponding to a particular outcome of the measurement. We now show how to replace $\hat{E}$ with a set of classical probability functions $R^{B}\left(\hat{E}|\alpha \right)$, one for each striation *B*. In keeping with the associated epistemic restriction, the function $R^{B}\left(\hat{E}|\alpha \right)$ will be constant along each line of *B*.

The definition of $R^{B}\left(\hat{E}|\alpha \right)$ is similar to the one in Equation (8).

(10) $$R^{B}\left(\hat{E}|\alpha \right)=\langle \psi_{\ell }\left|\hat{E}\right|\psi_{\ell }\rangle ,$$

where *ℓ* is again the unique line in *B* that contains α. Informally, we think of $R^{B}\left(\hat{E}|\alpha \right)$ as the probability of the outcome $\hat{E}$ when the system is at the point α (an illegal quantum state). Note that this function has a different normalization from the classical probability distributions describing a preparation. We can think of the uniform distribution over phase space—with the value $1/d^{2}$ for each point α—as representing the completely mixed state. (This interpretation comes from the discrete Wigner function). Therefore, we expect the following normalization:

(11) $$\begin{array}{cc} \; & \sum_{\alpha }\left[R^{B}\left(\hat{E}|\alpha \right)\times \frac{1}{d^{2}}\right]=\mathrm{tr}\left[\hat{E}\left(\hat{I}/d\right)\right]=\frac{1}{d}\mathrm{tr}\hat{E}\\ \Rightarrow & \sum_{\alpha }R^{B}\left(\hat{E}|\alpha \right)=d\;\mathrm{tr}\hat{E}. \end{array}$$

One can see from Equation (10) that the function is indeed normalized in this way.

We now turn our attention to the case of a transformation. In general, the quantum description of a normalization-preserving transformation is given by a completely positive, trace-preserving map, which in turn can be specified by a set of Kraus operators. We will replace this description by a set of classical probability distributions. We restrict our attention to operations that preserve the Hilbert-space dimension, and we restrict our attention to *unital* transformations, that is, transformations that leave the completely mixed state unchanged.

We begin by defining a set of *transition quasiprobabilities* that characterize a given transformation. For an operation E, these are defined by:

(12) $$Q_{E}\left(\beta |\alpha \right)=\frac{1}{d}\mathrm{tr}\left[{\hat{A}}_{\beta }E\left({\hat{A}}_{\alpha }\right)\right].$$

In particular, if E is a unitary transformation, we have:

(13) $$Q_{E}\left(\beta |\alpha \right)=\frac{1}{d}\mathrm{tr}\left[{\hat{A}}_{\beta }\hat{U}{\hat{A}}_{\alpha }{\hat{U}}^{†}\right].$$

In a discrete-Wigner-function formulation, we can interpret $Q_{E}\left(\beta |\alpha \right)$ as the quasiprobability that a system at the point α will move to the point β when the transformation E is applied. Thus, if the transformation is applied to a system described by the Wigner function $Q(\alpha |\hat{w})$, the resulting Wigner function $Q(\beta |E\left(\hat{w}\right))$ is given by:

(14) $$Q\left(\beta |E\left(\hat{w}\right)\right)=\sum_{\alpha }Q_{E}\left(\beta |\alpha \right)Q\left(\alpha |\hat{w}\right).$$

Though $Q_{E}\left(\beta |\alpha \right)$ plays the role of a probability in this equation, it is not a probability since it can take negative values [57]. It is, however, normalized as a probability distribution: $\sum_{\beta }Q_{E}\left(\beta |\alpha \right)=1$. Because our transformations are unital, $Q_{E}$ is also normalized over its second argument: $\sum_{\alpha }Q_{E}\left(\beta |\alpha \right)=1$.

We use $Q_{E}\left(\beta |\alpha \right)$ to define our classical transition probabilities (which are indeed nonnegative). Again, the framework for a transformation is specified by a symplectic transformation *S* chosen from the set T defined above. In the framework *S*, the probability that a system at point α will move to β is given by:

(15) $$R_{E}^{S}\left(\beta |\alpha \right)=\frac{1}{d^{2}}\sum_{\mu }Q_{E}\left(S\mu +\delta |\mu \right),$$

where δ=β−Sα. That is, we obtain $R_{E}^{S}\left(\beta |\alpha \right)$ simply by averaging $Q_{E}\left(\beta |\alpha \right)$ over the displacement class δ in which the ontic transition α→β lies. By definition, then, $R_{E}^{S}\left(\beta |\alpha \right)$ is constant over each displacement class.

What is much less obvious is that $R_{E}^{S}\left(\beta |\alpha \right)$ is always nonnegative. This was proven in [7], and we do not repeat the proof here. (For the special case d=2, the nonnegativity *depends* on using the special subgroup T of symplectic matrices. For odd primes, $R_{E}^{S}$ is nonnegative for any symplectic *S*). We also showed in that paper how to reconstruct the quantum operation E from the entire set of $R_{E}^{S}$’s.

We now have all the ingredients we need for a classical description of a whole experiment, within a specified framework. Let us suppose the experiment consists of a preparation, followed by a transformation, followed by a measurement. In terms of standard quantum mechanical concepts, we can compute the probability of a particular outcome via the equation:

(16) $$P\left(\hat{E}|E,\hat{w}\right)=\mathrm{tr}\left[\hat{E}E\left(\hat{w}\right)\right],$$

where $\hat{w}$ is the initial density matrix, E is the transformation, and $\hat{E}$ is the POVM element representing the outcome.

Within the classical framework $\left(B^{\prime },S,B\right)$, we can try to compute the same probability by writing:

(17) $$P\left(\hat{E}|E,\hat{w}\right)\overset{?}{=}\sum_{\alpha \beta }R^{B^{\prime }}\left(\hat{E}|\beta \right)R_{E}^{S}\left(\beta |\alpha \right)R^{B}\left(\alpha |\hat{w}\right).$$

Note that we are combining the probabilities in the standard way. Again, every function inside the sum is nonnegative and properly normalized, so the resulting probability is at least a legitimate probability. However, it is not the correct value. This is largely because the classical story associated with a specific framework is by no means the whole story. We need the predictions obtained from *all* the coherent frameworks in order to recover the quantum prediction. We show how this is done in the following subsection.

### 2.3. Recovering the Quantum Prediction: Summing the Nonrandom Parts

The formula for reconstructing the quantum prediction from the whole set of classical predictions is quite simple. As we noted in the Introduction, it depends on the concept of the “nonrandom part” of a probability, which we now explain.

For a probability distribution R(α) over the discrete phase space, we define the nonrandom part ΔR(α) to be the deviation from the uniform distribution:

(18) $$\Delta R\left(\alpha \right)=R\left(\alpha \right)-\frac{1}{d^{2}}.$$

For the probability of a measurement outcome $\hat{E}$, we define the nonrandom part by subtracting the probability we would assign to the outcome $\hat{E}$ if we were starting with the completely mixed state, or in phase-space language, if we were starting from the uniform distribution over phase space. Thus, we have:

(19) $$\Delta R^{B}\left(\hat{E}|\alpha \right)=R^{B}\left(\hat{E}|\alpha \right)-\frac{1}{d^{2}}\sum_{\gamma }R^{B}\left(\hat{E}|\gamma \right),$$

or, for an expression using standard quantum mechanical terms,

(20) $$\Delta P\left(\hat{E}|\hat{w}\right)=P\left(\hat{E}|\hat{w}\right)-\frac{1}{d}\mathrm{tr}\hat{E}.$$

For all these cases, “Δ” means that we are subtracting the “random part” of the given probability, that is, the value we would assign to the probability under a condition of minimal knowledge.

Let us now consider an experiment consisting of a preparation $\hat{w}$, a transformation E, and a measurement, one of whose possible outcomes is $\hat{E}$. We showed in [7] that we recover the quantum mechanically predicted probability of the outcome $\hat{E}$ via the following formula:

(21) $$\Delta P\left(\hat{E}|E,\hat{w}\right)=\sum_{F}\Delta P^{F}\left(\hat{E}|E,\hat{w}\right),$$

where the sum is over all coherent frameworks $F=(B^{\prime },S,B)$, and:

(22) $$P^{F}\left(\hat{E}|E,\hat{w}\right)=\sum_{\alpha \beta }R^{B^{\prime }}\left(\hat{E}|\beta \right)R_{E}^{S}\left(\beta |\alpha \right)R^{B}\left(\alpha |\hat{w}\right).$$

That is, within each framework, we compute the probability of $\hat{E}$ in an utterly standard way. What is nonstandard is that we then combine these various classical predictions by summing the nonrandom parts.

One component of the derivation of Equation (21) is the formula that inverts Equation (15), which was also proven in [7]:

(23) $$\Delta Q_{E}\left(\beta |\alpha \right)=\sum_{S}\Delta R_{E}^{S}\left(\beta |\alpha \right).$$

We will find this equation useful in Section 4 below.

## 3. The Composition Rules and Their Role as Foundational Postulates

In the preceding section, we started with the standard quantum mechanical description of each component of an experiment and then defined our classical probability functions in terms of the associated quantum concepts. Our ultimate aim, though, is to develop a self-contained formulation of quantum theory, in which the basic objects are epistemically restricted classical probability functions. This means that we cannot rely on the standard concepts of quantum theory to determine which sets of classical probability functions are allowed. We must find criteria that are independent of the vectors and operators of Hilbert space. It is to this aim that we now turn our attention. In this section, therefore, we switch to an operational understanding of the symbols *w*, E, and *E*. We use those symbols to refer to a preparation, a transformation, and a measurement outcome—processes and events one can observe in a lab—and not to any particular mathematical objects. The absence of hats on the symbols is a notational indication of this switch.

Again, the calculation in Equation (22), in which we compute the probability of the outcome *E* from the perspective of one of our classical observers, is quite ordinary—all the probabilities are being used in the standard way. It is only when we combine the classical predictions, via Equation (21), that we combine probabilities in a way that we would never do classically—by summing the nonrandom parts. We are inclined, then, to regard the summing of the nonrandom parts as the essentially quantum mechanical component of our formulation. We do not claim to fully understand the significance of this procedure. However, it does seem to capture what is quantum mechanical about our formalism, just as the superposition principle can be understood as the quintessential quantum mechanical feature of the usual formulation. We do not mean to imply that our rule is in any way an expression of the superposition principle, but only that we are giving our rule a fundamental status in the mathematical formalism. (The superposition principle is quite foreign to our approach, since we are working only with probabilities and not with amplitudes).

This circumstance leads us to ask whether the procedure of summing nonrandom parts can be used as a foundational principle, which could determine which sets of probability distributions are permitted.

To that end, let us consider the following four equations—the “composition rules”—all of which follow from the definitions of the preceding section, but all of which also make sense without reference to any Hilbert-space concepts. In these equations, the symbol Δ is consistently used to indicate the nonrandom part of whatever follows it:

1. Combining a preparation with a transformation to obtain another preparation:
  (24) $$\Delta R^{B^{\prime }}\left(\beta |E\left(w\right)\right)=\sum_{\left\{\left(S,B\right)|SB=B^{\prime }\right\}}\Delta \left[\sum_{\alpha }R_{E}^{S}\left(\beta |\alpha \right)R^{B}\left(\alpha |w\right)\right];$$
2. Combining two transformations in sequence to obtain another transformation:
  (25) $$\Delta R_{E_{2}\circ E_{1}}^{S}\left(\gamma |\alpha \right)=\sum_{\left\{\left(S_{2},S_{1}\right)|S_{2}S_{1}=S\right\}}\Delta \left[\sum_{\beta }R_{E_{2}}^{S_{2}}\left(\gamma |\beta \right)R_{E_{1}}^{S_{1}}\left(\beta |\alpha \right)\right];$$
3. Combining a transformation with a measurement outcome to obtain another measurement outcome:
  (26) $$\Delta R^{B^{\prime }}\left(E^{\prime }|\alpha \right)=\sum_{\left\{\left(B,S\right)|S^{-1}B=B^{\prime }\right\}}\Delta \left[\sum_{\beta }R^{B}\left(E|\beta \right)R_{E}^{S}\left(\beta |\alpha \right)\right],$$
  where $E^{\prime }$ is the measurement outcome that is equivalent to applying E and then obtaining the outcome *E*;
4. Combining a preparation *w* with a measurement outcome *E* to obtain the probability P(E|w) of the outcome *E* given the preparation *w*:
  (27) $$\Delta P\left(E|w\right)=\sum_{B}\Delta \left[\sum_{\alpha }R^{B}\left(E|\alpha \right)R^{B}\left(\alpha |w\right)\right].$$
  We can summarize all of these equations by saying that in any combination of the components of an experiment, one always sums the nonrandom parts of the classically expected results, the sum being over all frameworks that are consistent with the framework of the resulting classical probability function (or, in the last case, all frameworks that are coherent).

The equations listed above are all correct as statements within quantum theory, but our question now is whether they are *sufficient* to pick out the allowed sets of *R* functions.

They may not be fully sufficient. These equations set conditions on the whole system of probability distributions, describing all the components of an experiment, and there could be trade-offs among these components. It is conceivable, for example, that by being more restrictive in what we allow for measurements, we can be more generous in what we allow for preparations. In this paper, we avoid some of this worry by making the following three auxiliary assumptions, but we are not certain whether all these auxiliary assumptions are necessary:

- For each preparation *w* with probability functions $R^{B}\left(\alpha |w\right)$, there is a corresponding measurement outcome *E* with probability functions $R^{B}\left(E|\alpha \right)=dR^{B}\left(\alpha |w\right)$, and the random part of $R^{B}\left(E|\alpha \right)$ is 1/d;
- For an invertible transformation E with probability functions $R_{E}^{S}\left(\beta |\alpha \right)$, the probability functions of the inverse are $R_{E^{-1}}^{S}\left(\beta |\alpha \right)=R_{E}^{S^{-1}}\left(\alpha |\beta \right)$;
- Every preparation consistent with Equation (27) and Assumption A is physically possible. Moreover, the complete system of preparations, transformations, and measurement outcomes must be *maximal*. That is, it should not be possible to add any other transformation or measurement outcome without violating Equations (24)–(27) or one of our assumptions.

Assumption A minimizes the likelihood of precisely the kind of trade-off we described above. Assumption B gives the most natural definition of the inverse in our formalism. The spirit behind Assumption C is that we are starting with a picture in which all properly normalized probability functions are allowed. The composition rules and the auxiliary assumptions are intended simply to restrict the set of such functions, and we do not want to restrict it more than necessary.

To see how a proposed set of *R* functions might run afoul of the composition rules and the auxiliary assumptions, suppose that for a single qubit, we were to say that there exists a preparation *w* such that for each striation *B*,

(28) $$R^{B}\left(\alpha |w\right)=\left\{\begin{array}{c} \frac{1}{2}\;\mathrm{if}\;\alpha \;\mathrm{lies}\;\mathrm{on}\;\mathrm{the}\;\mathrm{ray}\;\mathrm{in}\;B\\ 0\;\mathrm{otherwise} \end{array}\right.$$

Then, by Assumption A, there exists a measurement outcome *E* such that:

(29) $$R^{B}\left(E|\alpha \right)=\left\{\begin{array}{c} 1\;\mathrm{if}\;\alpha \;\mathrm{lies}\;\mathrm{on}\;\mathrm{the}\;\mathrm{ray}\;\mathrm{in}\;B\\ 0\;\mathrm{otherwise} \end{array}\right.$$

These functions are perfectly consistent with the epistemic constraint—each is constant along each line of its striation *B*—but they are not consistent with Equation (27): the computed probability for the outcome *E*, given the preparation *w*, comes out to be two, which is not a legitimate value for a probability. Our question is whether similar considerations will rule out all other sets of *R*’s that do not correspond to legitimate quantum states and processes.

We have by no means answered this question in general, but we do have some answers for the special case of a single qubit. They are the subject of the following section.

## 4. The Case of a Single Qubit

Here, we show how Equations (24)–(27) and Assumptions A, B, and C apply to the case d=2.

### 4.1. The Allowed Preparations and Measurements

For now, let us continue to take *d* as any prime number. Let the functions $R^{B}\left(\alpha |w\right)$ describe a preparation *w*. Then, by Assumption A, there is a measurement outcome *E* described by $R^{B}\left(E|\alpha \right)=dR^{B}\left(\alpha |w\right)$. Inserting this *w* and *E* into Equation (27), we obtain:

(30) $$\Delta P\left(E|w\right)=\sum_{B}\Delta \left[\sum_{\alpha }R^{B}\left(E|\alpha \right)R^{B}\left(\alpha |w\right)\right].$$

Each term in this equation that is preceded by Δ is a probability assigned to the outcome *E*. Therefore, the Δ tells us to subtract 1/d (as is also specified in Assumption A). Collecting the constant terms on the right-hand side, we have:

(31) $$P\left(E|w\right)=\sum_{B,\alpha }\left[R^{B}\left(E|\alpha \right)R^{B}\left(\alpha |w\right)\right]-1.$$

Now, we replace $R^{B}\left(E|\alpha \right)$ with $dR^{B}\left(\alpha |w\right)$ to obtain:

(32) $$P\left(E|w\right)=d\sum_{B,\alpha }R^{B}{\left(\alpha |w\right)}^{2}-1.$$

In order to prevent P(E|w) from being larger than one, we need to insist that:

(33) $$\sum_{B,\alpha }R^{B}{\left(\alpha |w\right)}^{2}\leq \frac{2}{d}.$$

This condition must hold for every prime *d*. As we now show, for the case of a single qubit, it completely defines the set of allowed preparations.

For a qubit, Equation (33) becomes simply:

(34) $$\sum_{B,\alpha }R^{B}{\left(\alpha |w\right)}^{2}\leq 1.$$

Suppose we have a set of functions $R^{B}\left(\alpha |w\right)$ satisfying this inequality. Let us define the quantities $r_{x}$, $r_{y}$, and $r_{z}$ as follows:

(35) $$\begin{array}{cc} \; & r_{x}=\sum_{\alpha }{\left(-1\right)}^{\alpha_{p}}R^{X}\left(\alpha |w\right)\\ \; & r_{y}=\sum_{\alpha }{\left(-1\right)}^{\alpha_{q}+\alpha_{p}}R^{Y}\left(\alpha |w\right)\\ \; & r_{z}=\sum_{\alpha }{\left(-1\right)}^{\alpha_{q}}R^{Z}\left(\alpha |w\right), \end{array}$$

where *X*, *Y*, and *Z* are the horizontal, diagonal, and vertical striations, respectively. These equations can be inverted to give:

(36) $$\begin{array}{cc} \; & R^{X}\left(\alpha |w\right)=\frac{1}{4}\left[1+{\left(-1\right)}^{\alpha_{p}}r_{x}\right]\\ \; & R^{Y}\left(\alpha |w\right)=\frac{1}{4}\left[1+{\left(-1\right)}^{\alpha_{q}+\alpha_{p}}r_{y}\right]\\ \; & R^{Z}\left(\alpha |w\right)=\frac{1}{4}\left[1+{\left(-1\right)}^{\alpha_{q}}r_{z}\right]. \end{array}$$

One can see from Equation (36) that:

(37) $$\sum_{B,\alpha }R^{B}{\left(\alpha |w\right)}^{2}=\frac{3}{4}+\frac{1}{4}\left(r_{x}^{2}+r_{y}^{2}+r_{z}^{2}\right).$$

Therefore, Equation (34) is equivalent to the condition that the vector $\vec{r}=\left(r_{x},r_{y},r_{z}\right)$ has length no greater than one.

From the definition of $R^{B}\left(\alpha |w\right)$ in Section 2, one can show that the *R*’s given in Equation (36) correspond to the density matrix $\hat{w}=\frac{1}{2}\left(I+\vec{r}\cdot \hat{\vec{\sigma }}\right)$, where $\hat{\vec{\sigma }}$ is the vector of Pauli matrices. We see, then, that the condition (34) is indeed sufficient to restrict the set of *R*’s to their proper range (that is, to the range $|\vec{r}|\leq 1$). Assumption C then tells us that the entire set of such preparations is allowed. In this way, we recover the Bloch sphere.

Do our assumptions also pick out the valid measurement outcomes? In the standard quantum formalism, we can characterize the allowed POVM elements $\hat{E}$ by the following condition: an operator $\hat{E}$ is a valid POVM element if and only if the quantity:

(38) $$P\left(E|w\right)=\mathrm{tr}\left(\hat{E}\hat{w}\right)$$

lies in the interval [0,1] for every density matrix $\hat{w}$. Now, Equation (27) is simply an expression of Equation (38) in our formalism. Therefore, the condition that the P(E|w) appearing in Equation (27) must be in the range [0,1] is equivalent to the quantum condition we have just stated. Equation (27) thus picks out the valid measurement outcomes, as long as we know what the valid preparations are. This we do know, as we have seen in the preceding paragraph.

### 4.2. The Allowed Invertible Transformations

Here, we aim to determine what set or sets of invertible transformations on a qubit are consistent with our assumptions.

We begin by noting that for any invertible transformation E, we can derive from Assumption B that $R_{E}^{S}\left(\beta |\alpha \right)$ is normalized over its second index, as well as its first. (As we noted earlier, the same is true for any unital transformation, a concept that still makes sense in our phase-space setting). We use this fact a few times in what follows.

Our next step is to derive the representation of the identity transformation $R_{I}^{S}\left(\beta |\alpha \right)$. For d=2, there is a valid preparation given by the following probability distributions:

(39) $$\begin{array}{c} R^{X}\left(\alpha |w\right)=R^{Y}\left(\alpha |w\right)=\frac{1}{4}\;\begin{array}{cc} 1 & 1\\ 1 & 1 \end{array} \end{array}\;,\;R^{Z}\left(\alpha |w\right)=\frac{1}{2}\;\begin{array}{cc} 1 & 0\\ 1 & 0 \end{array}\;.$$

(The bottom left box of such a phase-space diagram corresponds to phase-space point α=(0,0), and the appropriate index increases by one when moving either up or right.) This corresponds to the spin-up state in the *z*-direction for a qubit. From the instance of Equation (24) that results from this preparation and the identity channel:

(40) $$\Delta R^{Z}\left(\beta |w\right)=\sum_{\left\{(S,B)|SB=Z\right\}}\Delta \left[\sum_{\alpha }R_{I}^{S}\left(\beta |\alpha \right)R^{B}\left(\alpha |w\right)\right].$$

Applying the normalization rule $\sum_{\alpha }R_{I}^{S}\left(\beta |\alpha \right)=1$ to the terms with B=X and B=Y, for which $R^{B}$ is uniform, yields a null contribution. What remains is:

(41) $$\Delta R^{Z}\left(\beta |w\right)=\Delta \left[\sum_{\alpha }R_{I}^{I}\left(\beta |\alpha \right)R^{Z}\left(\alpha |w\right)\right].$$

For this to hold true, the transitions of $R_{I}^{I}\left(\beta |\alpha \right)$ must not take either of the points on the nonzero line of $R^{Z}\left(\alpha |w\right)$ away from that line. However, this same argument can be made for a preparation corresponding to any other line and the form of $R_{I}^{I}\left(\beta |\alpha \right)$ should not change. This implies:

(42) $$R_{I}^{I}\left(\beta |\alpha \right)=\delta_{\alpha \beta }.$$

Therefore, whenever the identity transformation is inserted into Equation (24), the LHS of the equation will always equal the term on the RHS that includes $R_{I}^{I}\left(\beta |\alpha \right)$. The other two terms—corresponding to the symplectic matrices R and L—must sum to zero, which can only be possible for all preparations when:

(43) $$R_{I}^{R}\left(\beta |\alpha \right)=R_{I}^{L}\left(\beta |\alpha \right)=\frac{1}{4}.$$

Equations (42) and (43) thus give us the representation of the identity transformation.

We now make use of Equation (25) specialized to the case of a transformation being combined with its inverse. Leveraging Assumption B, we find:

(44) $$\begin{array}{c} \Delta R_{I}^{S}\left(\gamma |\alpha \right)=\sum_{S^{\prime }}\Delta \left[\sum_{\beta }R_{E}^{SS^{\prime }}\left(\gamma |\beta \right)R_{E}^{S^{\prime }}\left(\alpha |\beta \right)\right]. \end{array}$$

We again use the fact that the sum of $R_{E}$ over its second argument is unity. From this, it follows that we can move the Δ on the right-hand side of Equation (44) to the factors inside the sum over β (see Appendix B of [7]):

(45) $$\begin{array}{c} \sum_{S^{\prime }}\Delta \left[\sum_{\beta }R_{E}^{SS^{\prime }}\left(\gamma |\beta \right)R_{E}^{S^{\prime }}\left(\alpha |\beta \right)\right]=\sum_{S^{\prime },\beta }\Delta R_{E}^{SS^{\prime }}\left(\gamma |\beta \right)\Delta R_{E}^{S^{\prime }}\left(\alpha |\beta \right). \end{array}$$

From Equation (23), we have that:

(46) $$Q_{E}\left(\gamma |\beta \right)=\frac{1}{4}+\sum_{S}\Delta R_{E}^{S}\left(\gamma |\beta \right).$$

Combining Equations (44)–(46) with the inverse rule and the form of $R_{I}^{I}\left(\beta |\alpha \right)$, one can show that the transition quasiprobabilities for any invertible transformation can be thought of as an orthogonal matrix:

(47)∑βQE(γ|β)QE(α|β)=∑β14+∑SΔRES(γ|β)14+∑S′ΔRES′(α|β)(48) =14+∑S,S′Δ∑βRESS′(γ|β)RES′(α|β)(49) =14+∑SΔRIS(γ|α)(50) =δαγ.

Although we ultimately want to know what sets of transition probabilities $R_{E}^{S}$ are allowed in our theory, the argument is less cumbersome if we work with the quasiprobabilities $Q_{E}$ temporarily. They are of course well defined in terms of the transition probabilities $R_{E}^{S}$ (by Equation (23)).

We can express the Wigner function Q(α|w) for a qubit as a four-component column vector ${\vec{Q}}_{w}$ on which a transition quasiprobability matrix $Q_{E}$ acts. Thus, Equation (14) becomes:

(51) $${\vec{Q}}_{w^{\prime }}=Q_{E}{\vec{Q}}_{w}\;,$$

where $w^{\prime }=E\left(w\right)$. We also know that $Q_{E}$ and its inverse both preserve normalization, which means that each row and each column of $Q_{E}$ sums to unity. Because of this, we can write:

(52) $$\Delta {\vec{Q}}_{w^{\prime }}=Q_{E}\Delta {\vec{Q}}_{w}\;,$$

where $\Delta \vec{Q}$ is defined through our usual Δ notation, that is, by subtracting 1/4 from each component. Now, define the following orthogonal matrix:

(53) $$M=\frac{1}{2}\left(\begin{array}{cccc} 1 & 1 & -1 & -1\\ 1 & -1 & -1 & 1\\ 1 & -1 & 1 & -1\\ 1 & 1 & 1 & 1 \end{array}\right).$$

Then, we have:

(54) $$\left(M\Delta {\vec{Q}}_{w^{\prime }}\right)=MQ_{E}M^{T}\left(M\Delta {\vec{Q}}_{w}\right).$$

Note that the last component of either $M\Delta {\vec{Q}}_{w}$ or $M\Delta {\vec{Q}}_{w^{\prime }}$ is zero due to normalization. Therefore, these vectors are confined to three dimensions. Meanwhile, the matrix $MQ_{E}M^{T}$ is still orthogonal. Moreover, it is block diagonal, consisting of a 3×3 block in the upper left and the number 1 in the lower right. Consequently, it is effectively a 3×3 orthogonal matrix acting on the three-dimensional space in which $M\Delta {\vec{Q}}_{w}$ can have nonzero components. Let us define $\hat{w}$ to be $\sum_{\alpha }Q\left(\alpha |w\right){\hat{A}}_{\alpha }$. This matrix has unit trace, so we can express it as $\hat{w}=\left(1/2\right)\left(I+\vec{r}\cdot \hat{\vec{\sigma }}\right)$ for some real vector $\vec{r}$. Then, one can show from the definition of ${\hat{A}}_{\alpha }$ that the three nonzero components of $M\Delta {\vec{Q}}_{w}$ are the components $r_{1}$, $r_{2}$, $r_{3}$ of $\vec{r}$. Thus, the fact that $Q_{E}$ is orthogonal implies that every reversible transformation can be thought of as a rotation of the Bloch sphere, possibly combined with a reflection.

We now have a set of invertible transformations that is more permissive than that of a qubit, since it includes the possibility of reflection. That is, it includes transformations represented by 3×3 orthogonal matrices with determinant −1. (In standard quantum terms, it includes antiunitary transformations.) However, not all of the negative-determinant transformations are allowed, as we now show.

One can always decompose a negative-determinant orthogonal transformation of the sphere into an inversion through the center followed by a rotation. We denote the inversion operation as Ω. To find $R_{\Omega }^{S}$, note that the phase point operators are defined using an expansion of Pauli operators and have unit trace, so they can be represented by points in the same three-dimensional space in which $\vec{r}$ lives. For example, ${\hat{A}}_{\left(0,0\right)}=\frac{1}{2}\left(I+\vec{r}\cdot \hat{\vec{\sigma }}\right)$, where $\vec{r}=\left(1,1,1\right)$, and more generally, we can write ${\hat{A}}_{\alpha }=\frac{1}{2}\left(I+{\vec{r}}_{\alpha }\cdot \hat{\vec{\sigma }}\right)$. Therefore, the inversion operation $\Omega ({\hat{A}}_{\alpha })$ is well defined: extra minus signs appear before the $\hat{X}$, $\hat{Y}$, and $\hat{Z}$ terms. We can then use Equations (12) and (15) to find:

(55) $$\begin{array}{cc} \; & R_{\Omega }^{I}\left(\beta |\alpha \right)=\frac{1}{2}-\delta_{\alpha \beta },\\ \; & R_{\Omega }^{R}\left(\beta |\alpha \right)=R_{\Omega }^{L}\left(\beta |\alpha \right)=\frac{1}{4}. \end{array}$$

$R_{\Omega }^{I}$ has negative values and, therefore, is not compatible with our formalism.

This does not yet rule out any of the other negative-determinant transformations. (It does, however, rule out the possibility of including even a single such transformation if all the rotations are allowed, since we could then construct Ω.) Again, all negative-determinant transformations can be written as an inversion followed by a rotation E, and we can use the combination rule in Equation (25) to show that:

(56) $$R_{E\circ \Omega }^{S}\left(\beta |\alpha \right)=\frac{1}{2}-R_{E}^{S}\left(\beta |\alpha \right).$$

It follows that we can only allow transformations described by E∘Ω, if the rotation E is represented by transition probabilities that never exceed 1/2. In Appendix A, we show that this leaves us with only twelve possible rotations that can be composed with inversion to give legal transition probabilities. Let us call them $E_{j}$. These include 90 degree right-hand rotations around each of the six cardinal directions and 180 degree rotations around each axis that forms a 45 degree angle with a pair of cardinal axes.

The twelve operations $E_{j}\circ \Omega$ effect the permutations of the four vectors ${\vec{r}}_{\alpha }$. (Recall that these vectors correspond to the four phase point operators and thus to the four points of phase space.) Composing these operations, we obtain a set of twelve rotations of the form $E_{j}\circ E_{k}$. Altogether, this gives us a set of positive- and negative-determinant transformations that correspond to the twenty-four ways one can permute the four phase point operators. Although this set of twenty-four is quite different from the set of reversible transformations of a qubit, it is intriguing that it is a nontrivial set of transformations that can easily be understood classically.

We thus have two possibilities for the set of transformations: (i) a continuous set consisting of all the rotations of the Bloch sphere—the set we were aiming for—or (ii) a finite set that can be understood as comprising all possible permutations of the four ontic states. Both sets are maximal in the sense that they cannot be augmented with any other transformations.

To summarize, it appears that our composition rules and auxiliary assumptions do not uniquely lead to qubit physics. Nonetheless, our simple setup does bring us remarkably close. At this point, the best we can do is to include another assumption such as the continuity of the set of transformations that would eliminate the finite set.

## 5. Conclusions

For a quantum system with a prime Hilbert-space dimension, we have a way of decomposing the quantum description of an experiment into a set of classical, epistemically restricted descriptions. For each of these classical descriptions, which consist of nothing but probability functions, we can imagine an observer using these functions to compute the probability of any given outcome of the experiment. For any given classical observer, this prediction will be a bad prediction, but we know how to combine the predictions of all the classical observers to recover the correct quantum mechanical probability: we sum the nonrandom parts.

However, this picture begins with the standard formulation of quantum theory. Our aim is to develop an alternative, self-contained formulation of quantum theory in which the classical descriptions are the primary mathematical entities. The formulation we seek would thus be based entirely on actual probability functions defined on phase space. To create such a formulation, we need a set of criteria for determining when a given probabilistic description of a preparation, a transformation, or a measurement outcome is legitimate. In this paper, we have presented and begun to explore a set of equations that might serve as the basis for such criteria. These equations—our four composition rules—can all be placed under the heading, “sum the nonrandom parts.” We have been led to this approach by the fact that this intriguing prescription is the only non-classical element of our formalism. We are wondering whether summing the nonrandom parts is a key to what is characteristically “quantum” about quantum theory, and we have speculated that it may play a fundamental role loosely analogous to that of the superposition principle in the standard formulation.

Summing the nonrandom parts is a strange way to combine probability distributions. It could easily lead to illegal probabilities if there were not some constraints on the probability distributions being combined. Therefore, simply by insisting that the probabilities computed via this prescription are legitimate, we are implicitly placing constraints on our classical probability distributions. This fact has led us to ask the question: Are those constraints, along with a set of intuitively plausible auxiliary assumptions, sufficient to define the structure of quantum theory?

We addressed this question for the case of a single qubit, with only reversible transformations, and we found that we can recover the usual quantum rules that determine what states are allowed and what transformations are allowed. (For the case of transformations, we need an assumption such as continuity to rule out a particular finite set of unitary and antiunitary transformations that is consistent with our other assumptions.)

However, the case of a single qubit is relatively simple. In our formalism, the set of allowed states is determined entirely by the condition that:

(57) $$\sum_{B,\alpha }R^{B}{\left(\alpha |w\right)}^{2}\leq 1.$$

For a general qudit, we have an analogous equation:

(58) $$\sum_{B,\alpha }R^{B}{\left(\alpha |w\right)}^{2}\leq \frac{2}{d}.$$

However, for d>2, this is not the *only* condition required for a state to be legitimate. Therefore, any argument from our composition rules is not likely to be as simple as the one we were able to use for a single qubit.

Once we permit ourselves the extra assumption that the set of transformations is continuous, the reversible transformations on a single qubit are also relatively simple. They are equivalent to the rotations in three dimensions. Therefore, we mainly needed to show that the matrix of transition quasiprobabilities, $Q_{E}\left(\beta |\alpha \right)$, is an orthogonal matrix. In higher dimensions, this matrix is again orthogonal, but other conditions must also be met in order to arrive at the unitary transformations.

However, we have by no means used all the information available in our composition rules (Equations (24)–(27)). Therefore, one can hope that these equations constitute a sufficient or nearly sufficient set of restrictions for arbitrary prime *d*.

Ultimately, we would like to extend our work to all Hilbert-space dimensions. In the analysis of [2], a system with composite dimension *d* is treated as a composite system. It would be natural for us to use the same strategy here. Thus, the phase space for a system with dimension six would be a four-dimensional space, the Cartesian product of $Z_{2}^{2}$ and $Z_{3}^{2}$. A framework for a preparation or a measurement outcome would consist of a striation $B_{2}$ of $Z_{2}^{2}$ and a striation $B_{3}$ of $Z_{3}^{2}$. In future work, we plan to use this factorization scheme to extend our treatment of preparations and measurements to arbitrary composite dimensions and, indeed, to arbitrary composite discrete systems. We see no obstacles there. The treatment of *transformations*, on the other hand, is more challenging. One can show that, if *S* ranges over all the 2×2 symplectic matrices with entries in $Z_{d}$, where *d* is composite, then the whole set of probability distributions $R_{E}^{S}\left(\beta |\alpha \right)$, defined in the natural way, does not contain the information needed to reconstruct the transition quasiprobabilities $Q_{E}\left(\beta |\alpha \right)$. (Moreover, factoring the group of symplectic transformations into groups associated with the prime factors of *d* does not change the information content of the *R*’s.) This fact does not imply that our formalism cannot be extended to composite dimensions, but it does mean that new ideas will be needed.

Of course we would also like to extend our “classical” treatment of quantum theory to the case of continuous phase space, the realm that is truly the domain of classical mechanics. The concepts of striations, displacements, and symplectic transformations are all sensible concepts for such a phase space. However, we anticipate challenges in finding the proper analogue of our notion of the sum of the nonrandom parts. For example, whereas the “random part” of a probability distribution over a discrete phase space is simply the uniform distribution, there is no such thing as a normalized uniform distribution over an infinite phase space. We plan to address this and related issues in future work.

Finally, it is interesting to ask whether our formalism lends itself to an ontological account of a quantum experiment. Each of our imagined classical observers would have no problem finding a realistic interpretation of their description of the experiment: the system is always at some location in phase space, and when a transformation occurs, the system jumps probabilistically to some other point. It is much more difficult, though, to find an ontological account that incorporates all the classical descriptions. This is not to say it cannot be done. If it is possible, it will certainly require expanding the picture beyond that of a stochastic process on phase space. It would also require making physical sense of the mathematical prescription to sum the nonrandom parts.

## Appendix A

Here, we prove that there is one possible set of transformations allowed within our scheme that includes a finite set of negative-determinant orthogonal transformations of the Bloch sphere.

Recall that we started with only our set of rules and assumptions and made no reference to Hilbert space. Using these, we found that the legal transformations can be understood as orthogonal transformations of the Bloch sphere. We now wonder what the possible rotations are that can be composed with the inversion operations Ω without generating a negative probability. We have seen that such a rotation must itself never generate a probability greater than 1/2 for any of its *R* values. We could compute the *R* values for the orthogonal transformation $Q_{E}$ directly from Equation (15). However, since we have established a correspondence between rotations of the sphere and unitary transformations, it is legitimate to use Equation (13), together with Equation (15), to compute these values.

The virtue of this strategy is that any unitary operation on a qubit can be expressed in a simple way:

(A1) $$\hat{U}=u_{0}\hat{I}+iu_{1}\hat{X}+iu_{2}\hat{Y}+iu_{3}\hat{Z},$$

where $\vec{u}=\left(u_{0},u_{1},u_{2},u_{3}\right)$ is a real four-vector with unit length. The set of functions $R_{E}^{S}$ (where E refers to this unitary transformation) holds twelve values that we can calculate using Equations (13) and (15). (For each of the three *S*’s, $R_{E}^{S}$ has sixteen entries, but remember that these are partitioned into four displacement classes, each of which holds a single value of $R_{E}^{S}$.) These values are listed in the following phase-space diagrams, where the label on the left is the symplectic transformation *S* and the phase-space points correspond to the value δ=β−Sα.

(A2) $$\begin{array}{ccc} I: & & \begin{array}{cc} u_{3}^{2} & u_{2}^{2}\\ u_{0}^{2} & u_{2}^{1} \end{array}\\ R: & \frac{1}{4}\times & \begin{array}{cc} {\left(u_{0}-u_{1}+u_{2}-u_{3}\right)}^{2} & {\left(u_{0}+u_{1}-u_{2}-u_{3}\right)}^{2}\\ {\left(u_{0}+u_{1}+u_{2}+u_{3}\right)}^{2} & {\left(u_{0}-u_{1}-u_{2}+u_{3}\right)}^{2} \end{array}\\ L: & \frac{1}{4}\times & \begin{array}{cc} {\left(u_{0}-u_{1}+u_{2}+u_{3}\right)}^{2} & {\left(u_{0}+u_{1}+u_{2}-u_{3}\right)}^{2}\\ {\left(u_{0}-u_{1}-u_{2}-u_{3}\right)}^{2} & {\left(u_{0}+u_{1}-u_{2}+u_{3}\right)}^{2} \end{array} \end{array}$$

Suppose for now that all the $u_{j}$’s are nonnegative. Again, we have already seen in the main text that in order to be composed with the inversion operator Ω, a transformation can have no value of $R_{E}^{S}\left(\beta |\alpha \right)$ greater than 1/2. Therefore, we must have the following three relations:

(A3) $$\begin{array}{cc} \; & u_{0}^{2}+u_{1}^{2}+u_{2}^{2}+u_{3}^{2}=1,\\ \; & u_{j}\leq \frac{1}{\sqrt{2}},\\ \; & u_{0}+u_{1}+u_{2}+u_{3}\leq \sqrt{2}, \end{array}$$

where the second line is from $R_{E}^{I}$ and the third line is from $R_{E}^{R}$. The argument is easier to see if we define $v_{j}=\sqrt{2}\;u_{j}$. Then, the conditions on $v_{j}$ are:

(A4) $$\begin{array}{cc} \; & v_{0}^{2}+v_{1}^{2}+v_{2}^{2}+v_{3}^{2}=2,\\ \; & v_{j}\leq 1,\\ \; & v_{0}+v_{1}+v_{2}+v_{3}\leq 2. \end{array}$$

Because each $v_{j}$ is no larger than one, $v_{j}^{2}\leq v_{j}$. However, then, the only way to satisfy the first and third conditions is to make each $v_{j}^{2}$*equal* to $v_{j}$. This means each $v_{j}$ must be either zero or one. Then, the first equation tells us that exactly two $v_{j}$’s are equal to one and the other two are equal to zero. Therefore, exactly two of the $u_{j}$’s are equal to $1/\sqrt{2}$ and the other two are equal to zero.

Of course, we also have to deal with the possibility that one or more of the $u_{j}$’s is negative. However, looking at the values of *R* in Equation (A2), we see that all possible combinations of the plus and minus signs appear in $R_{E}^{R}$ and $R_{E}^{L}$. Therefore, we can replace the last *v* condition with $|v_{0}\left|+\right|v_{1}\left|+\right|v_{2}\left|+\right|v_{3}|\leq 2$. The middle equation can be $|v_{j}|\leq 1$. Then, the same argument applies.

The only option we are left with is to form four-vectors where only two entries are $\pm 1/\sqrt{2}$ and the other two are zero. There are twenty-four ways to do this, but one-half of that set of vectors is just the negative of the other half. Mapping back from $\vec{u}$ to $\hat{U}$, this minus sign is just a phase that can be ignored. We are left with twelve possible rotations compatible with the inversion operator.
